# Enrichment of homoacetogens converting H_2_/CO_2_ into acids and ethanol and simultaneous methane production

**DOI:** 10.1002/elsc.202200027

**Published:** 2022-12-07

**Authors:** Yaxue He, Chiara Cassarini, Piet N.L. Lens

**Affiliations:** ^1^ National University of Ireland Galway Galway Ireland

**Keywords:** acetogenesis, anaerobic sludge, H_2_/CO_2_ fermentation, methanogenesis, solventogenesis

## Abstract

An anaerobic granular sludge was enriched to utilize H_2_/CO_2_ in a continuous gas‐fed up‐flow anaerobic sludge reactor by applying operating conditions expected to produce acetic acid, butyric acid, and ethanol. Three stages of fermentation were found: Stage I with acetic acid accumulation with the highest concentration of 35 mM along with a pH decrease from initial 6 to 4.5. In Stage II, H_2_/CO_2_ was replaced by 100% H_2_ to induce solventogenesis, whereas butyric acid was produced with the highest concentration of 2.5 mM. At stage III with 10 µM tungsten (W) addition, iso‐valeric acid, valeric acid, and caproic acid were produced at pH 4.5–5.0. In the batch tests inoculated with the enriched sludge taken from the bioreactor (day 70), however, methane production occurred at pH 6. Exogenous 15 mM acetate addition enhanced both the H_2_ and CO_2_ consumption rate compared to exogenous 10, 30, and 45 mM acetate by the enriched sludge. Exogenous acetate was failed to be converted to ethanol using H_2_ as electron donor by the enriched acetogens.

**Abbreviations**
ADH: alcohol dehydrogenase
CO: carbon monoxide
CO_2_
: carbon dioxide
EtOH: ethanol
FDH: formate dehydrogenase
H_2_
: hydrogen
HAc: acetic acid
TS: total solid
VFAs: volatile fatty acids
VS: volatile solid
W: tungsten
WLP: Wood–Ljungdahl pathway

## 1 INTRODUCTION

CO_2_ fermentation to generate bio‐commodities (e.g., acetic acid [1]) or biofuels (e.g., ethanol [2] and methane [3] relieves the paradox of fossil fuel utilization and carbon emission reduction. CO_2_ bioconversion mitigates carbon emission and hence becomes a promising economical and sustainable way for biofuel production [4, 5].

CO_2_ can be converted to VFAs (volatile fatty acids) and alcohols via two‐stage fermentation in the Wood–Ljungdahl pathway (WLP) by autotrophic acetogens including *Clostridium spp*. The first stage is acetogenesis with accumulation of acetic acid, followed by solventogenesis under stress conditions such as nutrient limitation or low pH [6]. The mechanism of solventogenesis, however, still remains to be explored. One of the widely recognized mechanisms to induce solventogenesis is a low pH [7]. Low pH (below 5) induces more undissociated acids that can enter the cells, which convert the acids to neutral charged ethanol to avoid their death caused by an intracellular pH drop [8]. On the other hand, microorganisms are one of the key components in CO_2_ autotrophic fermentation, for example, *Clostridium autoethanogenum* [9] and *Clostridium carboxidivorans* [10, 11]. Several pure strains have been studied, however, mixed culture fermentations are easier to implement at a large scale than pure cultures with the merits of resistance to non‐sterile conditions [12]. The potential products converted from H_2_ and CO_2_ include:

(1) $$\begin{array}{ccc} & & 72{\mathrm{CO}}_{2}\left(g\right)+4H_{2}\left(g\right)\to {\mathrm{CH}}_{3}\mathrm{COOH}\left(l\right)+2H_{2}O\left(l\right)\\ & & \;▵G^{\theta }=-75.4{\;\mathrm{kJ}\;\mathrm{mol}}^{-1} \end{array}$$

(2) $$\begin{array}{ccc} & & 2{\mathrm{CO}}_{2}\left(g\right)+6H_{2}\left(g\right)\to {\mathrm{CH}}_{3}{\mathrm{CH}}_{2}\mathrm{OH}\left(l\right)+3H_{2}O\left(l\right)\\ & & \;▵G^{\theta }=-96.5{\;\mathrm{kJ}\;\mathrm{mol}}^{-1} \end{array}$$

(3) $$\begin{array}{ccc} & & {\mathrm{CO}}_{2}\left(g\right)+4H_{2}\left(g\right)\to {\mathrm{CH}}_{4}\left(g\right)+2H_{2}O\\ & & \;▵G^{\theta }=-130.4{\;\mathrm{kJ}\;\mathrm{mol}}^{-1} \end{array}$$

(4) $$\begin{array}{ccc} & & {\mathrm{CH}}_{3}\mathrm{COOH}\left(l\right)+2H_{2}\left(g\right)\to {\mathrm{CH}}_{3}{\mathrm{CH}}_{2}\mathrm{OH}\left(l\right)+H_{2}O\left(l\right)\\ & & \;▵G^{\theta }=-25.2{\;\mathrm{kJ}\;\mathrm{mol}}^{-1} \end{array}$$

Limited studies reported ethanol production from H_2_/CO_2_ [13, 14]. The positive role of exogenous acetate on ethanol production by a *Clostridium* strain has been reported using syngas as the gaseous substrate [15]. However, whether acetic acid with H_2_ can be directly converted to ethanol by mixed cultures remains to be explored. Therefore, one possible strategy for enhancing solventogenesis is to supply exogenous acetate with H_2_ as the electron donor under low pH by mixed cultures.

Tungsten (W) is an important trace element involved in the formation of enzyme activity such as formate dehydrogenase (FDH), one of the key enzymes in the WLP, converting CO_2_ into formate. It has been reported that FDH synthesis could be stimulated in the presence of W [16]. The other key metalloenzyme related to W is alcohol dehydrogenase (ADH) catalyzing the reduction of acetyl CoA to ethanol [17]. Tungsten can enhance ethanol production from carbon monoxide (CO) by anaerobic granular sludge [18].

This study investigated CO_2_ and H_2_ fermentation by heat‐treated granular sludge in a bioreactor with both gas and medium circulation at 25°C. It was assumed that ethanol production could be enhanced by feeding 100% H_2_ or tungsten from acetic acid produced by homoacetogens. Acetate produced from H_2_/CO_2_ or pure H_2_ as the gaseous substrate and ethanol degradation were further investigated in batch tests by the enriched sludge taken from the reactor after 70 days of operation, from which the homoacetogenesis, methanogenesis and solventogenesis potential was assessed.

**HIGHLIGHT**

- H_2_/CO_2_ replaced by 100% H_2_ stimulated butyric acid and ethanol production at pH 4.5–5 by anaerobic sludge.
- 10 µM tungsten addition enhanced caproic acid production at pH 4.0–4.5.
- Methane was mainly produced from H_2_/CO_2_ and exogenous acetate and ethanol at pH 6 and 25°C by the enriched sludge.
- Enriched sludge failed to convert acetate and 100% H_2_ to ethanol.

**Practical application**

CO_2_ fermentation simultaneously mitigates carbon emission and generates valuable bioenergy products and hence becomes a promising economical and sustainable way of biofuel production. This study investigated CO_2_ and H_2_ fermentation by heat‐treated granular sludge in a bioreactor with both gas and medium circulation at 25°C. H_2_/CO_2_ replaced by 100% H_2_ stimulated butyric acid and ethanol production at pH 4.5–5 by anaerobic sludge. 10 µM tungsten addition enhanced caproic acid production at pH 4.0–4.5. Methane was mainly produced from H_2_/CO_2_ and exogenous acetate and ethanol at pH 6 and 25°C by the enriched sludge. Exogenous 15 mM acetate addition enhanced both the H_2_ and CO_2_ consumption rate compared to exogenous 10, 30, and 45 mM acetate by the enriched sludge. This study provides an attractive strategy in acetogenesis and solventogenesis in a bioreactor via 100% H_2_ and tungsten addition by anaerobic sludge and obtained methane production in H_2_/CO_2_ fermentation by the enriched sludge.

## 2 MATERIALS AND METHODS

### 2.1 Biomass and medium composition

The same inoculum anaerobic granular sludge from a wastewater treatment plant was used as in our previous study on acids and alcohol production from H_2_/CO_2_ [2]. The total solid (TS) and volatile solid (VS) content was 42.7 (± 1.0) g L^−1^ and 24.8 (± 0.5) g L^−1^, respectively. The granular sludge was first centrifuged at 5500 rpm for 10 min to remove the supernatant and the pellet was heat‐treated at 90°C for 15 min to select for spore forming acetogens as described by Dessì et al. [19]. The medium was prepared according to a previous study [2].

### 2.2 Experimental set‐up

#### 2.2.1 Semi‐continuous gas fed bioreactor

**FIGURE 1:**
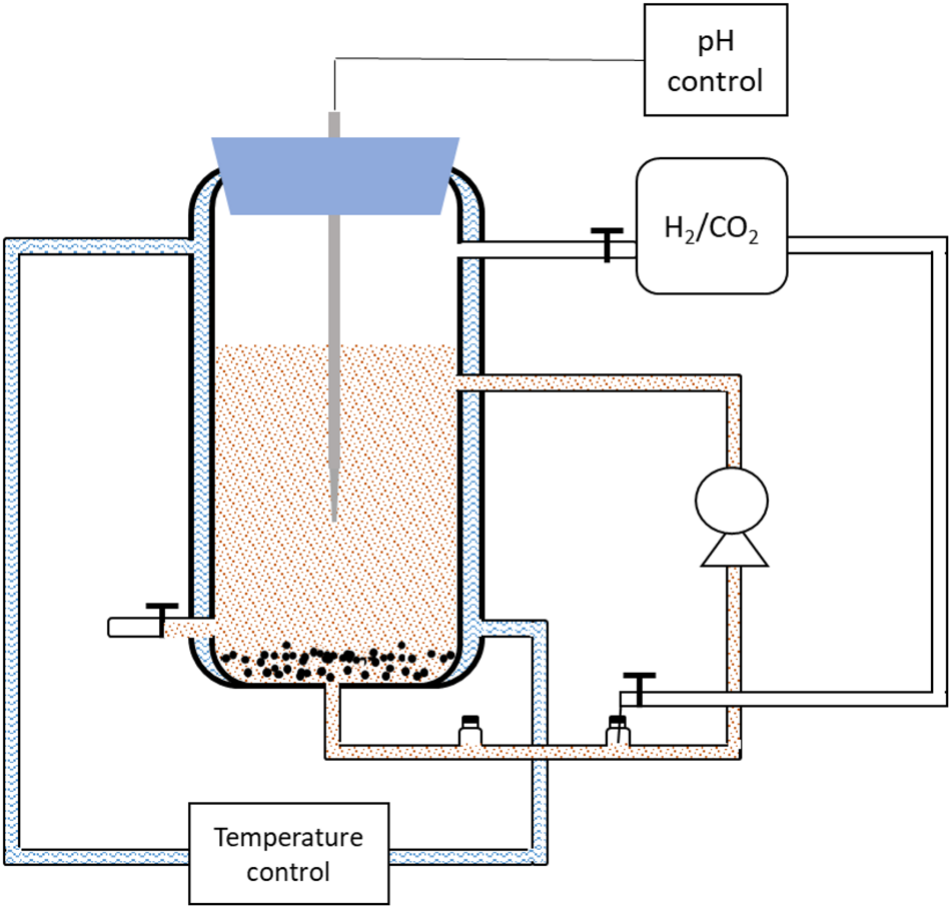
Diagram of the up‐flow gas reactor with pH control

An up‐flow semi‐continuous gas fed reactor was set‐up with a total working volume of 1 L (Figure 1) and liquid flow rate was 60 ml min^−1^ by a Verdeflex pump (Utrecht, The Netherland). A 10 L gas bag filled with H_2_/CO_2_ (80/20 v/v) was connected on the gas outlet. H_2_/CO_2_ gas was cycled at a gas flow rate of 10 ml min^−1^ controlled by gas tight tubes using a Verdeflex pump (Utrecht, The Netherland) and a mass flow meter (FMA‐1618A, Omega, San Antonio, US). The temperature was controlled at 25°C by a water jacket. The initial pH was 6.0 and when the pH decreased to 4.5, the pH control system would start working to prevent a further pH drop by adding 1 M NaOH to stimulate solventogenesis.

#### 2.2.2 Batch tests

Batch experiments were conducted in 120 ml serum bottles with 50 ml medium and 5% enriched sludge (day 70). The bottles were sealed with rubber stoppers and capped with aluminum crimp caps. All bottles were pressurized with pure H_2_ or H_2_/CO_2_ (80/20 v/v) at an initial pressure of 1.8 bar and were incubated at 150 rpm and at 25°C.

### 2.3 Experimental design

#### 2.3.1 Semi‐continuous gas fed bioreactor operation

The semi‐continuous gas fed bioreactor operation included three stages. In stage I (0–26 d), the reactor was fed with H_2_/CO_2_ gas (80/20 v/v) with the initial pH of 6.0 for acetic acid production without pH control. In stage II (day 27–50), H_2_/CO_2_ was replaced by 100% H_2_ to stimulate ethanol production at a pH controlled at 4.5‐5. In stage III (day 50–70), 10 µM tungsten was added to the medium to stimulate solventogenesis according to the report of Chakraborty et al. [18], while the gas phase was still 100% H_2._

Microbial community analysis was conducted for the anaerobic granular sludge in duplicate (G‐a, G‐b) on 10 ml bioreactor suspension samples at the end of stages I, II, and III (in triplicate, III‐a, b, and c). At the end of the stage I (day 26, the log phase of the autotrophic acetogens) to sustain and further enrich the sludge, 10 ml liquid sludge from the reactor was inoculated into two 120 ml batch bottles with 50 ml liquid medium (duplicate). H_2_/CO_2_ (80/20, v/v) was used as the substrate and the initial pH was 6.0. The bottles were incubated at 150 rpm and at 25°C in a water‐bath shaker.

#### 2.3.2 Batch studies on solventogenesis using different substrates by enriched sludge

To elucidate the conversion pathway and failure of solventogenesis in the reactor, batch tests of H_2_/CO_2_, 15 mmol L^−1^ acetate+ H_2_/CO_2_ and 10 µM tungsten+ H_2_/CO_2_ were conducted using the bioreactor sludge as the inoculum. The enriched sludge from gas fed reactor after 70 days fermentation was used as the inoculum for the following batch tests. To investigate the effect of exogenous acetate on ethanol production using H_2_ as electron donor, the bottles were sparged with 100% H_2_ and H_2_/CO_2_ (v/v, 80/20) and 5% inoculum at an initial pressure 1.8 bar. Acetate was added to make the final concentration of 10, 15, 30, and 40 mmol L^−1^, respectively. To test whether acetic acid and ethanol degradation occurred in the reactor, 15 mmol L^−1^ acetate + 5 mmol L^−1^ ethanol, 30 mmol L^−1^ acetate + 15 mmol L^−1^ ethanol were added with H_2_/CO_2_ (v/v, 80/20) in the headspace in batch tests using 5% enriched sludge.

### 2.4 Analysis

#### 2.4.1 Gas phase

H_2_, CO_2_, and CH_4_ concentrations were measured using a HP 6890 gas chromatograph (GC, Agilent Technologies, Palo Alto, USA) equipped with a thermal conductivity detector (TCD). The GC was fitted with a 15‐m HP‐PLOT Molecular Sieve 5A column (ID 0.53 mm, film thickness 50 mm). The oven temperature was kept constant at 60°C. The temperature of the injection port and the detector was maintained constant at 250°C. Helium was used as the carrier gas.

#### 2.4.2 VFAs and solvents analysis

Volatile fatty acids, ethanol, and butanol concentrations were analyzed for each bottle from the liquid phase (1 ml) using high performance liquid chromatography (Agilent Co., Palo Alto, USA) equipped with a refractive index detector (RID) and an Agilent Hi‐Plex H column (Internal diameter × length, 7.7 × 300 mm, size 8 µM). A H_2_SO_4_ solution (5 mM) was used as mobile phase at a flow rate of 0.7 ml min^−1^ and with a sample injection volume of 50 µl. The column temperature was set at 60°C and the RID detector at 55°C.

#### 2.4.3 Microbial analysis

DNA was extracted using a DNeasy PowerSoil Kit (QIAGEN, Germany) following the manufacturer's protocol. Approximately 0.5 g of the solids from the samples was used for DNA extraction. The extracted DNA was quantified and its quality was checked by a Nanodrop 2000c Spectrophotometer (Thermo Scientific, USA). A total of 1,103,482 sequences were obtained from all investigated samples (Table S1). After eliminating chimeras, a sequence identity of 70%, across at least 80% of the representative sequences, was a minimal requirement for considering reference sequences. Further processing of the operational taxonomic units (OTUs) and taxonomic assignments were performed using the QIIME software package (version 1.9.1, http://qiime.org/). Abundances of bacterial taxonomic units were normalized using lineage‐specific copy numbers of the relevant marker genes to improve estimates [20].

## 3 RESULTS

### 3.1 Enrichment of acetogenic sludge and production of acids and ethanol in gas fed reactor

**FIGURE 2:**
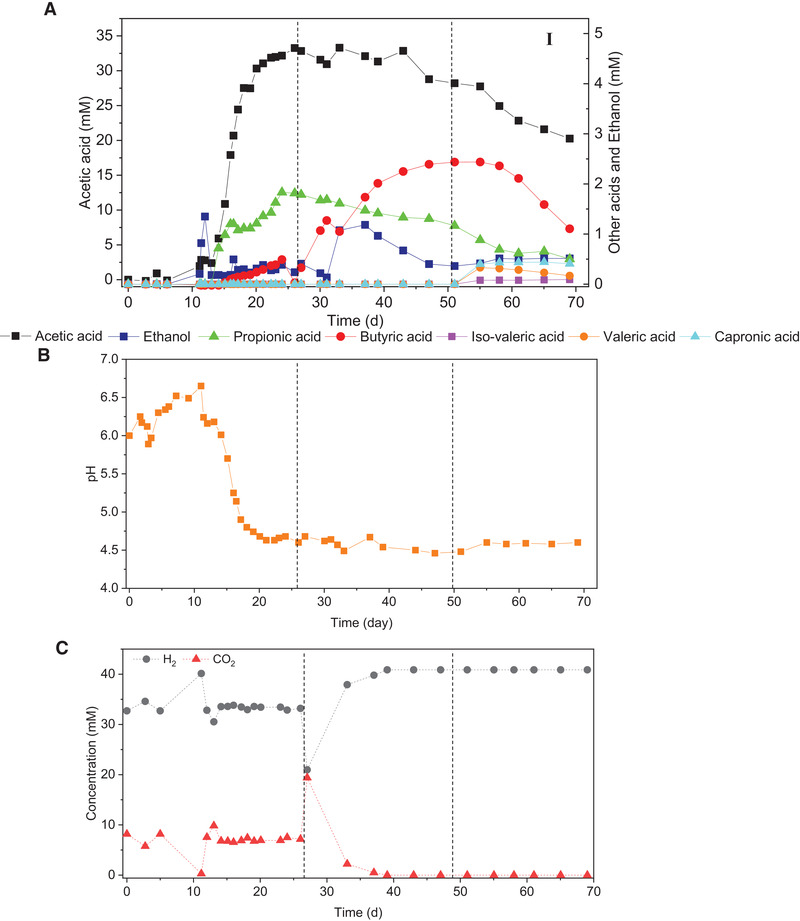
H_2_/CO_2_ fermentation in a semi‐continuous gas fed reactor by anaerobic granular sludge. (A) acids and ethanol production, (B) change of pH and (C) H_2_, CO_2_ concentration from H_2_/CO_2_ or H_2_ by granular sludge. The substrate of stages I, II and III are, respectively, H_2_/CO_2,_ H_2_ and H_2_ + 10 µM tungsten

During the reactor operation, after 10 days of adaption, acetic acid started to be produced and reached to 35 mM (Figure 2A, Equation 1). Ethanol was detected at day 11 and increased to 1.35 mmol L^−1^ at day 12 but it was then degraded (Figure 2A, Equation 2). Instead, butyric acid started to be produced at day 12 when ethanol degradation occurred and increased to 0.5 mmol L^−1^ at day 26. Propionic acid started to be produced at day 14 and reached to 1.82 mmol L^−1^ at day 26. The pH was decreased along with the accumulation of acetic acid and kept at 4.5–5.0 after day 21 (Figure 2B). However, ethanol production was not observed when the pH was as low as 4.5 from day 21 to 26 (Figure 2A).

To stimulate ethanol production from acetic acid, H_2_/CO_2_ was replaced by 100% H_2_ at day 27 (stage II, 27–50 d). Indeed, 100% H_2_ addition induced ethanol production and it reached to 1.2 mmol L^−1^ at day 37 (Figure 2A, Equation 4). Thereafter, ethanol production started to decrease to a concentration of 0.5 mmol L^−1^ (Figure 2A). Meanwhile, butyric acid accumulated and reached 2.4 mmol L^−1^ (Figure 2A). After feeding 100% H_2_, both ethanol and butyric acid production occurred from day 30 to 37, but butyric acid kept increasing along with the consumption of ethanol from day 37 to 50. The concentration of both acetic acid and propionic acid decreased at the end of the stage II.

At stage III, 10 µM tungsten addition induced both acetic acid and butyric acid degradation, accompanied with the production of valeric acid and caproic acid, respectively, 1.3 and 0.4 mmol L^−1^ at the end of incubation (Figure 2A).

### 3.2 Effect of exogenous acetate and tungsten on H_2_/CO_2_ conversion by enriched sludge

**FIGURE 3:**
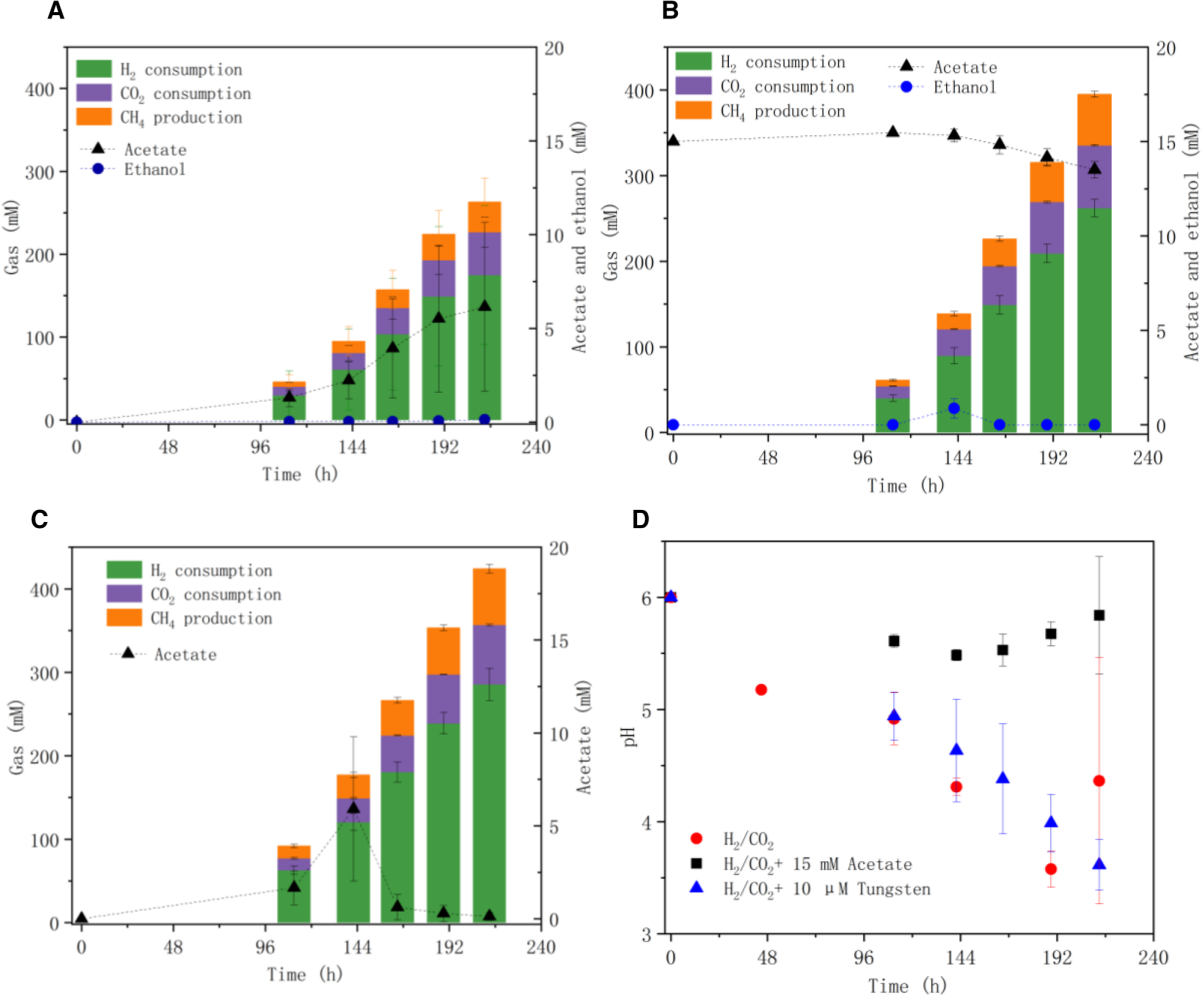
H_2_ and CO_2_ consumption and CH_4_, acetate and ethanol production by enriched sludge sampled day 70 from the bioreactor using (A) H_2_/CO_2_, (B) H_2_/CO_2_ + 15 mM acetate and (C) H_2_/CO_2_ + 10 µM tungsten as the substrate and (D) change of pH in these incubations

When using H_2_/CO_2_ as the substrate (the control) for the enriched sludge (day 70), acetic acid was produced with a final concentration of 6.1 mmol L^−1^ (Figure 3A, Table S2). Methane production was observed along with the acetic acid production and 36.6 mmol L^−1^ methane had accumulated at the end of the incubation (Figure 3A). H_2_ and CO_2_ consumption was, respectively, 160 and 40.6 mmol L^−1^ at the end of the incubation (Figure 3A).

With 15 mmol L^−1^ acetate addition, 0.9 mmol L^−1^ ethanol was produced after 144 h but it was degraded after 192 h and did not accumulate at the end of the incubation. However, methane production was observed and accumulated to 60.0 mmol L^−1^ at the end of the incubation. The acetic acid concentration slightly decreased from initially 15 to 13 mmol L^−1^ at the end of the incubation (Figure 3B). H_2_ and CO_2_ consumption was, respectively, 262.0 and 73.3 mmol L^−1^ at the end of the incubation (Figure 3A) and was correspondingly 1.6‐ and 1.8‐fold higher than the control to which no external acetate was provided. The H_2_ and CO_2_ consumption rate increased to, respectively, 0.85 and 0.26 mmol L^−1^ h^−1^ compared to the control of 0.59 and 0.18 mmol L^−1^ h^−1^ (Table S2).

The addition of 10 µM tungsten enhanced the H_2_ and CO_2_ consumption of 285.8 and 71.1 mmol L^−1^, respectively, at a H_2_ and CO_2_ consumption rate of 1.02 and 0.25 mmol L^−1^ h^−1^, respectively, compared to the control. Methane (67.6 mmol L^−1^) was produced at the end of the incubation (Figure 3C). The methane production was from acetate as the substrate since the produced acetic acid at 144 h (6.0 mmol L^−1^) was almost totally consumed in the 10 µM W+H_2_/CO_2_ incubation upon completion of the experiment (Figure 3C, D). Surprisingly, the pH of the control and the 10 µM tungsten incubation decreased quickly even below 4 after 192 h but methane production was still detected (Figure 3D).

### 3.3 Effect of exogenous acetate on H_2_/CO_2_ conversion by enriched sludge

**FIGURE 4:**
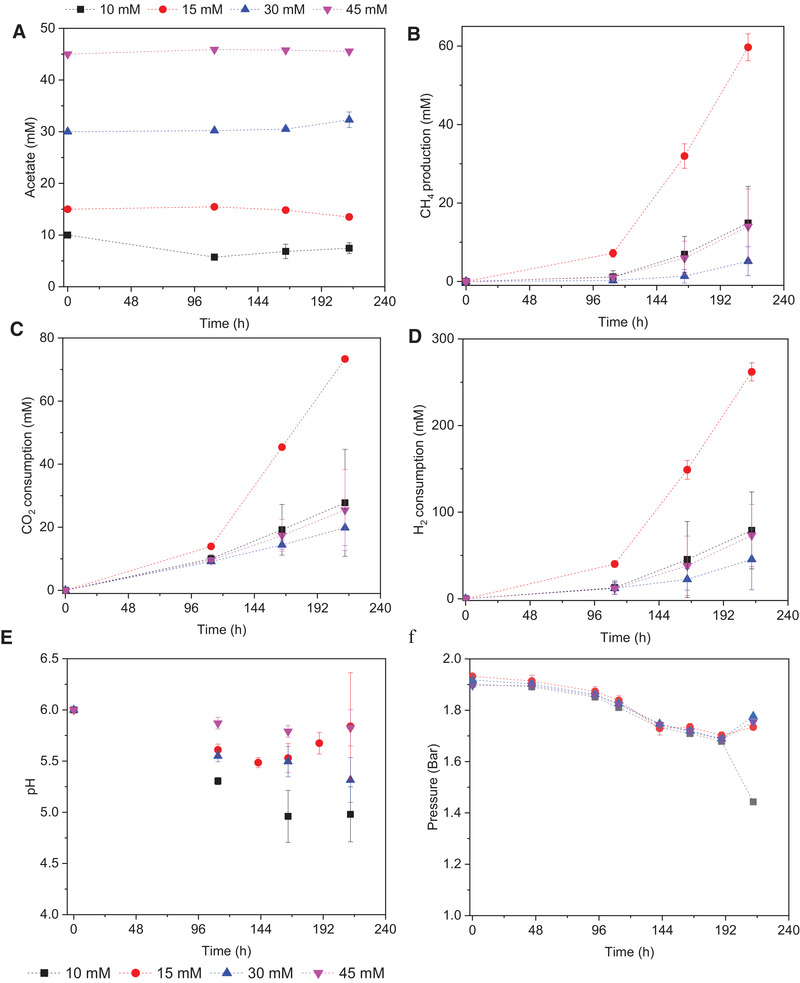
Effect of 10, 15, 30 and 45 mM exogenous acetate on production profiles by enriched sludge day 70 bioreactor operation using H_2_/CO_2_ as the substrate (A) acetate concentration, (B) CH_4_, c) CO_2_ and (D) H_2_ production, (E) change of pH and (F) gas pressure

Initially, acetate was not significantly consumed while it slightly increased at the initial concentration of 15 and 30 mmol L^−1^ acetate (Figure 4A). Methane production reached to 14.9, 59.7, 5.2, and 14.0 mmol L^−1^ along with the increased initial 10, 15, 30, and 45 mmol L^−1^ acetate concentration. Correspondingly, CO_2_ consumption was respectively, 27.7, 73.3, 19.8 and 25.4 mmol L^−1^, whereas the H_2_ consumption amounted to 78.9, 261.9, 45.4, and 73.1 mmol L^−1^, respectively. Correspondingly, the pH decreased from initial 6 to 5.0–5.2 at both initial 15 and 30 mmol L^−1^ acetate due to the positive net acetic acid production (Figure 4E). The gas pressure was decreased slowly during the incubation, because part of the gas pressure came from the methane production (Figure 4F).

The 15 mM acetate addition reached the highest CH_4_ production, CO_2_, and H_2_ consumption compared with 10, 30, and 45 mmol L^−1^ acetate, while the acetic acid concentration slightly decreased at the end (Figure 4B–D). 10 and 50 mmol L^−1^ acetate had a similar effect on CH_4_ production and H_2_ and CO_2_ consumption, while supplementing 30 mmol L^−1^ acetate obtained the lowest CH_4_ production, CO_2_ and H_2_ consumption (Figure 4B–D).

Further experiments demonstrated that when using 100% H_2_ and in the absence of CO_2_, the ethanol production process did not happen after 240 h incubation. The pH did not change during the incubation and the gas pressure did not decrease. The failure of acetate and H_2_ utilization might be because the enriched acetogens were mostly autotrophic acetogens, which was further confirmed by the microbial community analysis (see below).

### 3.4 Acetate and ethanol conversion in the presence of H_2_/CO_2_ by enriched sludge

Acetate and ethanol were added to simulate the conversion process, that is, the reverse β oxidation pathway, to further assess if longer chain VFAs were produced, as observed in the reactor. With 15 HAc + 5 EtOH and 30 HAc + 15 EtOH, methane production was observed and reached, respectively, 54.1 and 46.3 mmol L^−1^. Neither ethanol nor longer chain fatty acids were produced during the incubation.

**FIGURE 5:**
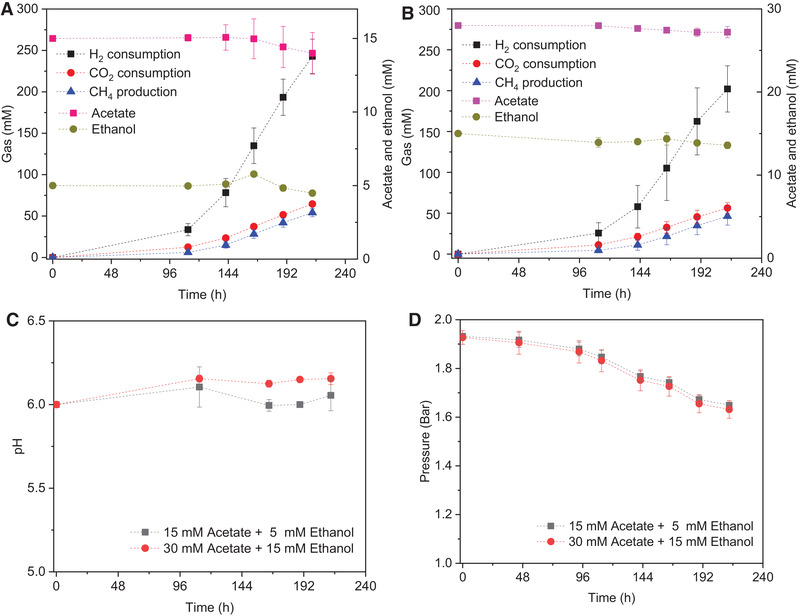
H_2_ and CO_2_ consumption and CH_4_, acetate and ethanol production in the presence of (A) 15 mM acetate + 5 mM Ethanol and (B) 30 mM Acetate + 15 mM Ethanol, (C) pH and (D) gas pressure change by enriched sludge sampled day 70 using H_2_/CO_2_ as the substrate

With 15 mmol L^−1^ acetate and 5 mmol L^−1^ ethanol addition, CH_4_ production and CO_2_ and H_2_ consumption were all higher compared to the incubations supplied with 30 mmol L^−1^ acetate and 15 mmol L^−1^ ethanol addition (Figure 5). Both the acetate and ethanol concentration slightly decreased during the incubation (Figure 5A,B). The pH was slightly increased possibly due to the decreased dissolved CO_2_ in the liquid medium induced by the consumption of headspace CO_2_ (Figure 5C). The gas pressure decreased slowly and showed a similar trend between the 15 HAc + 5 EtOH and 30 HAc + 15 EtOH groups.

The enriched sludge was further checked for the addition of glucose to possibly enhance the biomass grow and mixotrophy. However, the ethanol production did not significantly enhance compared to the solely glucose fed incubation (Figure S1).

### 3.5 Microbial analysis

**FIGURE 6:**
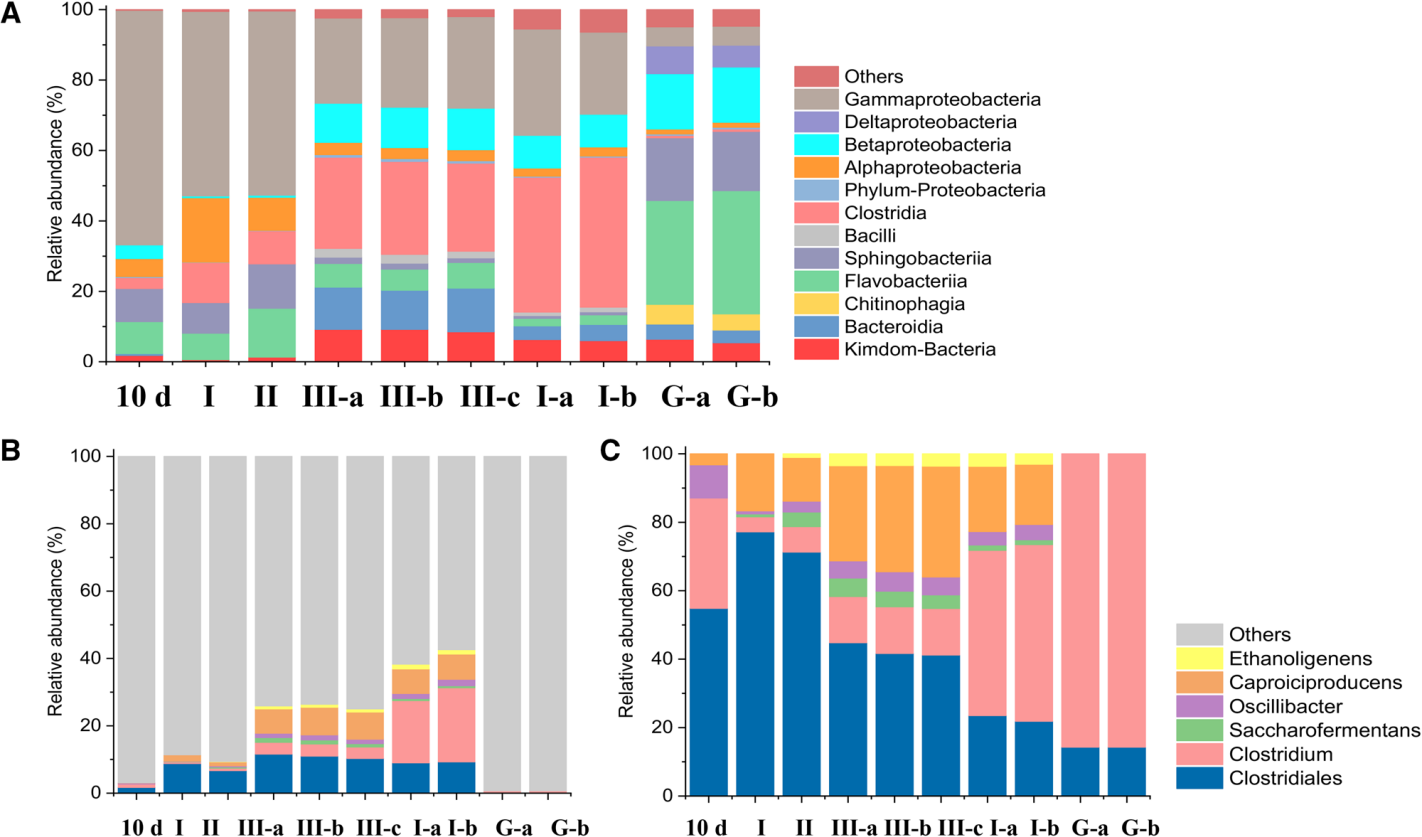
Relative abundance of microorganism from suspended sludge at 10 d, and the end of stages I, II, III (III‐a, b and c are triplicates) and the granular sludge inoculum (G‐a, G‐b) at genus level. The two batch bottles used the sludge taken from the bioreactor as inoculum (stage I, day 26) (I‐a, I‐b). (A) Microbial analysis of all bacteria, (B) Genus level in the *Clostridiales* order, the relative abundance is relative to all the bacteria, and (C) Genus level in the *Clostridiales* order, the relative abundance is relative to the *Clostridiales* order

Microbial analysis of the suspended sludge of the bioreactor showed the relative abundance of acetogens related at class level *Clostridia*. On day 10, when the acetic acid started to be produced, they comprised a relative abundance of 3.1%, it increased to 11.4% at the stage II and 9.4% at the stage III, finally reaching about 25–26% (Figure 6A).

For the *Clostridium* genus, the relative abundance with 0.1% at day 10 increased to, respectively, 0.5% at stage II, 0.7% at stage III and about 3.5% at the end of each stage (Figure 6B). Figure S2 shows the acetic acid and ethanol production in the batch bottles inoculated with enriched sludge from the bioreactor at day 70. The microbial analysis data (I‐a, I‐b of bottle 1, 2, respectively) showed that the higher relative abundance of the *Clostridium* genus compared to the reactor sludge sampled on day 10. *Clostridium* was enriched with a relative abundance of 3.5% in the bioreactor (day 70) and increased to 18.5 and 22.0 % in the enriched batch bottle 1 and 2 (Figure 6B).

Figure 6C shows the distribution of the *Clostridium* genus. The *Clostridium* genus and other acetogens belonging to the *Clostridia* class occupied above 60% at the end of the incubation (III‐a, b, c) (Figure 6C). In the *Clostridia* class, the relative abundance of the *Caproiciproducens* genus increased from 3.2% on day 10 to about 30% at the end of the incubation. The increase and enrichment of *Caproiciproducens* was corresponding to the increased caproic acid production at the end of the incubation. Small amounts of the *Ethanoligenens* genus were enriched with around 3% at the end of the incubation (triplicates, III‐a, b, c), which might have contributed to the ethanol production process during the fermentation. The *Oscillibacter* genus existed during the whole fermentation process with a relative abundance of 9.8% at day 10, then decreased to 5.8% at the end of the incubation (Figure 6C). *Oscillibacter* is known to be involved in acidogenesis during dark fermentation [21] and this microorganism might play a role in the acetic acid accumulation during the adaption stage.

## 4 DISCUSSION

### 4.1 VFAs and ethanol production by anaerobic granular sludge in the gas fed reactor

This study showed that 100% H_2_ addition induced both butyric acid and ethanol production, while 10 µM tungsten induced caproic acid production at a pH as low as 4.5–5.0. Ethanol production was observed during the H_2_/CO_2_ fermentation process and 100% H_2_ as electron donor, but it was subsequently degraded. Considering the inoculum applied was an undefined mixed culture, ethanol has been degraded to acetic acid in the presence of CO_2_ (Equation 5) or used as the electron donor for butyric acid production (Equation 6).

(5) $$\begin{array}{ccc} & & 2{\mathrm{CH}}_{3}{\mathrm{CH}}_{2}\mathrm{OH}\left(l\right)+2{\mathrm{CO}}_{2}\left(g\right)\to 3{\mathrm{CH}}_{3}\mathrm{COOH}\left(l\right)\\ & & \;▵G^{\theta }=-32.2{\;\mathrm{kJ}\;\mathrm{mol}}^{-1} \end{array}$$

(6) $$\begin{array}{ccc} & & {\mathrm{CH}}_{3}{\mathrm{CH}}_{2}\mathrm{OH}\left(l\right)+{\mathrm{CH}}_{3}\mathrm{COOH}\left(l\right)\\ & & \;\to {\mathrm{CH}}_{3}{\mathrm{CH}}_{2}{\mathrm{CH}}_{2}\mathrm{COOH}\left(l\right)+H_{2}O \end{array}$$

The first ethanol degradation (day 11) was possibly due to its oxidation to acetic acid in the presence of CO_2_ since butyric acid production was insignificantly observed at that time (Figure 2A). The second ethanol decrease (day 37–50) possibly supplied butyric acid production via the reverse β oxidation pathway [22], during which the butyric acid concentration increased along with the ethanol consumption (Figure 2).

The presence of CO_2_ on ethanol utilization could have induced formation of longer chain fatty acids. Roghair et al. [23] reported butyric acid and caproic acid production via controlling the ethanol use under different CO_2_ loading rates (0.5 and 2.5 LCO_2_ L^−1^ d^−1^) by anaerobic granular sludge. However, our previous study using the same anaerobic granular sludge demonstrated that the ethanol oxidation to acetic acid was priority over chain elongation in the presence of CO_2_ at initial pH 5.7 and 6.5 by the same anaerobic granular sludge [24]. H_2_ might have acted as electron donor for the chain elongation process, which has been reported in the literature [25]. Moreover, tungsten enhanced the chain elongation process at pH 4.0–4.5. Caproic acid production occurred at a pH as low as 4, which has been seldom reported since chain elongation processes generally occur at high pH value [7]. On the other hand, acetogens adding a carbon to the carbon chain could be enriched after the second ethanol degradation and further contributed to the chain elongation process. Further research with ^13^C NMR and labelled substrate (e.g., CO_2_, ethanol, and acetate) is required to elucidate the biochemical conversions in the sludge.

### 4.2 Methane was the main by‐product during chain elongation process when pH increased to 6 at 25°C by enriched sludge

This study showed that, with gaseous H_2_/CO_2_, 15 mM acetic acid addition reached the highest methane production, CO_2_ and H_2_ consumption compared to the 0, 10, 30, and 45 mmol L^−1^ acetic acid addition by enriched sludge (day 70) (Figure 3A,B, Figure 4). Despite of the different extent of gas consumption, methane occupied the main product of the enriched sludge. An initial pH of 6 could be attributed to the methane production in batch tests by the enriched sludge while methane production was totally inhibited at pH 4.5–4.7 in the bioreactor. The inhibited methane production in the reactor could be attributed to the heat pre‐treatment and the long‐time operation at low pH of 4.5. However, along with the operation, methanogens could be enriched in the inoculum although the production of methane can be inhibited at a pH of 4.5 [11]. Although methane production can be inhibited when the pH was lower than 6, its production has been observed in a few reactors operating at low pH, especially along with increased operation time [26]. A mixed bacterial culture isolated from slurry incubations even produced methane from H_2_/CO_2_ at acid pH lower than 4 [27, 28]. Another reason might be the gas feeding mode or different mass transfer rate between 1 bar gas pressure in the reactor, whereas an initial 1.8 bar in the batch bottles. Higher gas pressure induced more CO_2_ dissolution in the medium and may stimulate hydrogenotrophic methanogens [23].

### 4.3 CO_2_ instead of exogenous acetate can be used for acetogenesis or methanogenesis by enriched sludge

This study showed that exogenous acetate with 10, 15, 30, and 45 mmol L^−1^ cannot be used for ethanol or methane production in the presence of 100% H_2_ by the enriched sludge. Even with H_2_/CO_2_ as the gaseous substrate, the maximum acetate consumption occupied 13.3% (thus 2 mmol L^−1^) in the 15 mmol L^−1^ acetate incubation. Ethanol (Equation 7) and methane (Equation 8) production from exogenous acetate failed using 100% H_2_ as electron donor by the enriched sludge. This might be because the enriched microorganisms after 70 days incubation in the bioreactor were autotrophic acetogens, such as the *Clostridia* and *Bacilli* class using CO_2_ instead of acetate as the substrate.

(7) $${\mathrm{CH}}_{3}\mathrm{COOH}+2H_{2}\to {\mathrm{CH}}_{3}{\mathrm{CH}}_{2}\mathrm{OH}+H_{2}O$$

(8) $${\mathrm{CH}}_{3}\mathrm{COOH}\to {\mathrm{CH}}_{4}+{\mathrm{CO}}_{2}$$

## 5 CONCLUSION

Autotrophic acetogens were enriched in a H_2_/CO_2_ gas fed reactor for acetic acid, butyric acid and caproic acid production from heat‐treated anaerobic granular sludge treating dairy wastewater. 100% H_2_ induced butyric acid and ethanol production at pH 4.5–5, but ethanol was degraded and might have contributed to the butyric acid production. 10 µM tungsten addition induced caproic acid production at pH 4.0–4.5. The *Clostridia* order was enriched at the end of the gas fed reactor and contributed to VFAs and ethanol production. The enriched sludge mainly produced methane from H_2_/CO_2_, exogenous acetate and ethanol in batch incubations at pH 6 and 25°C. The enriched sludge failed to convert acetate and 100% H_2_ to ethanol at an initial pH of 6.

## AUTHOR CONTRIBUTIONS

Yaxue He: Conceptualization, methodology, formal analysis, investigation, writing – original draft, and visualization. Chiara Cassarini: Conceptualization, software, data curation, writing – review & editing Flora Marciano, and investigation. Piet N.L. Lens: Project administration, resources, supervision, funding acquisition, and writing – review & editing.

## CONFLICT OF INTEREST

The authors declare that the research was conducted in the absence of any commercial or financial relationships that could be construed as a potential conflict of interest.

## Supporting information

Supporting Information

## Data Availability

The datasets used and/or analyzed during the current study are available from the corresponding author on reasonable request.
