# Supplementary material for: Enrichment of homoacetogens converting H2/CO2 into acids and ethanol and simultaneous methane production
Source: Eng Life Sci. 2022 Dec 7;23(2):e2200027. doi: 10.1002/elsc.202200027 (PMC9893759; doi:10.1002/elsc.202200027)
Supplement: Supplementary file 1 — Supporting Information [file ELSC-23-e2200027-s001.docx]

# **Supporting Information**

# Enrichment of homoacetogens converting H_2_/CO_2_ into acids and ethanol and simultaneous methane production

Yaxue He*, Chiara Cassarini, Piet N. L. Lens

National University of Ireland Galway, University Road, H91 TK33, Galway, Ireland

**SI Table 1** The operational taxonomic units (OTUs) sequence table per sample statistics for microbial community analysis

| **Sample** | **Input sequences** | **Sequences assigned to OTUs** | **Sequences assigned to taxa** | **Count after lineage-specific copy-number correction** | **Median sequence length after preprocessing** |
| --- | --- | --- | --- | --- | --- |
| **1.V3V4a** | 72702 | 66109 | 66109 | 19727 | 424 |
| **2.V3V4a** | 58761 | 53699 | 53699 | 17182 | 424 |
| **3.V3V4a** | 63815 | 57402 | 57402 | 18719 | 424 |
| **4a.V3V4a** | 59467 | 52177 | 52177 | 15680 | 422 |
| **4b.V3V4a** | 58181 | 51113 | 51113 | 15304 | 422 |
| **4c.V3V4a** | 56605 | 49821 | 49821 | 15285 | 422 |
| **4d.V3V4a** | 174983 | 126305 | 126305 | 30873 | 402 |
| **4e.V3V4a** | 175270 | 134029 | 134029 | 32259 | 402 |
| **5.V3V4a** | 73013 | 61246 | 61246 | 30388 | 424 |
| **6.V3V4a** | 168634 | 137257 | 137257 | 61726 | 424 |
| **7.V3V4a** | 56950 | 48158 | 48158 | 20268 | 424 |
| **8a.V3V4a** | 163540 | 136572 | 136572 | 74150 | 422 |
| **8c.V3V4a** | 160028 | 129594 | 129594 | 73452 | 422 |
| **TOTAL** | 1341949 | 1103482 | 1103482 | 425013 | 419 |

**SI Table 2** The highest production of acetic acid and ethanol, CH_4_ accumulation, H_2_ and CO_2_ consumption at the end of incubation by enriched sludge in batch tests.

| **Compound** | **H_2_/CO_2_** | **H_2_/CO_2_+ 15 mM Hac** | **H_2_/CO_2_+ 10 μM W** |
| --- | --- | --- | --- |
| Acetic acid (mM) | 6.1±4.5 | 15.5±0.1 | 5.9±3.4 |
| Ethanol (mM) | 0.1±0.1 | 0.9 ±0.5 | 0 |
| CH_4_ (mM) | 36.6 ± 28.6 | 59.7 ± 3.4 | 67.6±5.2 |
| H_2_ consumption (mM) | 174.8±84.1 | 262.0 ± 10.4 | 285.5±19.4 |
| CO_2_ consumption (mM) | 51.7±18.2 | 73.3 ± 1.0 | 71.1±1.0 |

**SI Fig. 1** The acetic acid and ethanol production of the two enriched bottles using enriched sludge from bioreactor (day 26, stage I) as inoculum.

a)

b)

**SI Fig. 2**

H_2_/CO_2_

H_2_ + Glucose

Glucose

H_2_/CO_2_ + Glucose

**SI Fig. 2** Effect of glucose (0.5 g/L) on H_2_/CO_2_ bioconversion by enriched sludge.
